# Human protozoa infection and dysplasia in ulcerative colitis: a neglected aspect in a prominent disease

**DOI:** 10.1007/s00436-023-07972-7

**Published:** 2023-09-15

**Authors:** Enas F. Abdel Hamed, Nahed E. Mostafa, Shaimaa M. Farag, Mohamed N. Ibrahim, Basma H. Ibrahim, Hayam E. Rashed, Mona Radwan, Salem Y. Mohamed, Ramy El Hendawy, Eman M. Fawzy

**Affiliations:** 1 Department of Medical Parasitology, Faculty of Medicine, Zagazig University, El Kawmia Square, Zagazig, Sharkia Governorate Egypt; 2 Clinical Laboratories Department, College of Applied Medical Sciences, Jouf University, Qurrayat, Kingdom of Saudi Arabia; 3 Department of Pathology, Faculty of Medicine, Zagazig University, Zagazig, Sharkia Egypt; 4 Department of Community and Occupational Medicine, Faculty of Medicine, Zagazig University, Zagazig, Sharkia Egypt; 5 Department of Internal Medicine, Gastroenterology & Hepatology Unit, Zagazig University, Zagazig, Egypt; 6 Department of Tropical Medicine, Zagazig University, Zagazig, Egypt

**Keywords:** Ulcerative colitis, Protozoal infections, Dysplasia, FC, AOPPs, MTs, p53Abs, PD-L1

## Abstract

The chance of getting colorectal cancer (CRC) is higher in people with chronic ulcerative colitis (UC). The impact of parasitic infections on UC is underappreciated. The purpose of this study was to look into the effect of intestinal protozoal infections on the dysplastic changes generated by UC. The research included 152 adult patients with histologically confirmed UC and 152 healthy controls. Fecal samples were examined for the presence of parasites and fecal calprotectin (FC). The enzyme-linked immunosorbent assay measured serum anti-p53 antibodies (p53Abs) and metallothioneins (MTs). The advanced oxidation protein products (AOPPs) and reduced glutathione (GSH) levels were measured by a spectrophotometric method in all subjects. Serum C-reactive protein (CRP) and IL-6 were also measured. In addition, histopathological and immunohistochemical investigations of intestinal tissue were done. Our results exhibited significant increases in FC and CRP, IL-6, AOPPs, MTs, and p53Abs in ulcerative colitis patients with parasitic infections compared to those without parasites. In contrast, GSH levels showed a significant decrease in the same group compared with other groups. Histopathological and immunohistochemical assessments of intestinal tissue signified severe inflammation and strong expression of PD-L1 in patients with parasitic infections compared to others without parasitic infections. Our research indicated a greater frequency of intestinal protozoa in UC patients with elevated inflammatory and dysplastic biomarker levels. This suggests that these parasites may be involved in the etiology of chronic UC and the associated carcinogenetic process. This is the first report of a link between parasitic infections and dysplastic alterations in UC patients.

## Introduction

Ulcerative colitis is a type of chronic inflammatory bowel disease (IBD) causing superficial damage to the mucosal layer of the colon and rectum (Ungaro et al. 2017). Chronic diarrhea and fecal blood are these patients’ most common clinical features. UC patients are expected to have a 2.4-fold greater CRC risk than the general population (Jess et al. 2012). Early onset IBD appears to be associated with an increased risk of CRC (Olén et al. 2020). In addition to CRP and FC, serum sIL-2R and IL-6 levels can be used to determine disease activity status in UC patients (Mavropoulou et al. 2020). Inhibition of inflammation has therapeutic benefits as it affects the steps of tumor development, including initiation, promotion, invasion, and metastasis (Romano et al. 2016). Biscaglia et al. (2021) reported that 25 IBD-CRC patients had their 39 genes implicated in cancer predisposition.

Over 450 million people are infected with intestinal parasites (Pestehchian et al. 2015). Infections with *Cryptosporidium* spp occurred more frequently in patients with colorectal cancer than in controls, regardless of age or gender (Sulżyc-Bielicka et al. 2018). Sawant et al. (2020) hypothesize an association between human cryptosporidiosis and colon cancer, while more than 20% of the world’s cancer burden is attributed to infectious pathogens. Colorectal cancer is the most common cancer linked to *Cryptosporidium* infection. In addition, it has been linked to an increased risk of *Cryptosporidium* spp. and *Blastocystis hominis* infections (Sulżyc-Bielicka et al. 2021; Taghipour et al. 2022).

Oxidative stress is an imbalance between prooxidants and antioxidants, intimately linked to inflammatory processes associated with the development and exacerbation of IBD (Tian et al. 2017). Advanced oxidation protein products are new oxidative stress protein markers with pro-inflammatory properties. Moreover, because AOPP accumulation promotes the development of IBD, so, it can be used as a non-invasive activation marker (Alagozlu et al. 2013).

Glutathione is one of the most prevalent thiol antioxidants in cells (Braidy et al. 2019). In addition, it has critical enzymatic defense mechanisms within the mucosa of colon that preserves proteins in their reduced form (Morgenstern et al. 2003). So, it protects cells from reactive oxygen species (ROS) connected to cancer development (Liu et al. 2018).

Every living organism contains metallothioneins, a class of tiny proteins involved in crucial biological processes such as cell replication and apoptosis (Cioffi et al. 2004). In pathological situations, such as different cancer kinds, serum MT levels are markedly elevated (Krizkova et al. 2010). Na et al. (2017) hypothesized the connection between metallothioneins and colon cancer as its development enhanced the expression of metallothioneins.

The most often reported somatic gene mutations in human cancer are p53 gene mutations, which increase p53 gene outputs in cancerous cells. This can trigger an immunological reaction by producing circulating anti-p53 antibodies (Hamouda et al. 2011). However, most TP53 mutations in CRC are missense mutations that compromise the function of wild-type p53. As a result, it boosts cancer cell stemness, proliferation, invasion, and metastasis, which aid in developing the disease (Liebl and Hofmann 2021).

This study aimed to look into the possible relationship between intestinal protozoal infections and the inflammatory and dysplastic alterations in ulcerative colitis.

## Subjects and methods

Our research was carried out at Zagazig University’s Department of Parasitology, Faculty of Medicine, from January 2021 to January 2022. It was authorized by the Medical Ethics Committee of Zagazig University’s Faculty of Medicine in compliance with the Helsinki Declaration. It was registered at the Institutional Review Board (IRB) #9855-9-1-2022. The patients and controls provided both informed and written permission. The Montreal categorization of the degree and severity of UC was used. Standard clinical, endoscopic, radiographic, and pathological criteria were used to confirm the diagnosis of UC (Silverberg et al. 2005). Procolitis, left sided colitis, and severe or pancolitis are all UC categories.

### Study population

This is a prospective case-control study that included 152 normal controls (GI) and 152 ulcerative colitis cases. Of the 152 cases with UC, 45 were free of parasitic infection (GII), and 107 were infected with parasites (GIII). During the initial enrollment visit, a gastroenterologist performed a full demographic and clinical exam for controls and patients.

#### Patients with IBD

Inclusion criteria: (i) For at least 3 months, IBD was diagnosed using the standard Montreal classification (Satsangi et al. 2006). (ii) IBD patients with ileocolic or colic locations. (iii) Patients ranging in age from 25 to 60.

Exclusion criteria: (i) Using antibiotics or any other probiotic bacterial supplement in the previous 3 months. (ii) Nonsteroidal anti-inflammatory medicines were used in the previous 3 months. (iii) A recent (within the last 3 months) diagnosis of gastrointestinal bacterial or parasite infections.

#### Controls (CTRLs)

Inclusion criteria: (i) Subjects with asymptomatic gastrointestinal disease (using a questionnaire to rule out any chronic conditions and present gastrointestinal complaints). (ii) Individuals under the age of 60 who get a colonoscopy to check for colorectal cancer. (iii) Absence of any macroscopic lesions including the diverticulae. (iv) Histological evaluation of intestinal biopsy samples obtained during colonoscopy revealed the absence of microscopic lesions.

Exclusion criteria: (i) Using antibiotics or any other probiotic bacterial supplement in the previous 3 months. (ii) Nonsteroidal anti-inflammatory medicines were used in the previous 3 months. (iii) A recent (within the last 3 months) diagnosis of gastrointestinal bacterial or parasite infections. (iv) Severe psychiatric disorder as the primary clinical concern. (v) Any other serious illnesses, as well as a history of drug or alcohol abuse.

### Sample collection

In the current study, three stool samples were collected from both control participants and UC patients for parasitological investigation to detect intestinal pathogenic protozoa. Controls and patients were instructed to bring a stool sample from their morning stool immediately after their initial consultation or throughout their inpatient stay for fecal calprotectin measurement, according to Bathe et al. (2019). Iodine-staining smears (King 1975) and the formalin-ethyl acetate concentration technique were used to identify protozoa in stool samples (Truant et al. 1981; Henricksen and Pohlenz 1981). For *Cryptosporidium parvum* (*C. parvum*) detection, a modified Ziehl–Neelsen technique was used. The stained smears were scanned for the presence of the parasite using the 100-oil immersion lens.

### Surveillance colonoscopy

All patients had a colonic biopsy conducted consistent with published American gastroenterological association recommendations for UC surveillance colonoscopy (Winawer et al. 2003). The formalin-fixed colon samples were sent for histological assessment by the pathologist. Throughout the endoscopy, serum samples were collected from each patient and stored at −80 °C until use.

### Measurement of serological biomarkers

(1) Serum AOPP level estimation according to Witko-Sarsat et al. (1998), (2) serum GSH level was detected by spectrophotometry, in which 5.5′-dithiobis (2-nitrobenzoic acid) is reduced by GSH, which results in the formation of a yellow molecule. A commercial kit (Bio Diagnostic, Egypt) was used to quantify the reduced chromogen’s absorbance at 405 nm, which is proportional to the GSH concentration (Sedlak and Lindsay 1968). (3) Determining the serum metallothionein level: After removing coexisting antibody-reactive protein using an acid (1 mol/L HCl) and heat (100 °C, 10 min) treatment, MTs in the blood were measured using an ELISA kit from RayBiotech, Inc. (Cousins 1991). (4) Serum p53Ab assay: p53Abs were identified using a commercially available ELISA kit (Quantikine) provided by Clinilab (Kirsch and Kastan 1998). The absorbance cutoff value was computed using the manufacturer’s instructions (cutoff = 26.3).

### The immunohistochemical study

The immunohistochemistry staining was carried out as follows: It was carried out using an automated immunostainer (Ventana BenchMark XT; USA). Sections were deparaffinized with xylene, rehydrated with alcohol, and saturated with 0.03% hydrogen peroxide, subsequently undergoing antigen retrieval. The main antibodies used were anti-p53, a mouse monoclonal antibody (Ab-6, clone DO-1, dilution 1:30, Thermo Scientific Lab Vision), and anti-PD-L1 in dilution of 1:100 (M3653, clone 22C3, Dako, Glostrup, Denmark). The ultraView Universal DAB Detection Kit was used. Finally, the sections were counterstained with hematoxylin and mounted in DPX. PD-L1 staining within the lamina propria was assessed separately and categorized according to the percentage of positive inflammatory cells within the entire area of the lamina propria as 0: negative, 1: 0.1–1%, 2: > 1–5%, 3: > 5–50%, and 4: > 50% PD-L1 (Jakubowska et al. 2016); P53 positive: strong staining to moderate staining in dysplastic cells; P53 negative: no staining or weakness or patchiness (Jung et al. 2014). PDL1 was diluted at a 1:100 ratio. PDL1 was diluted as (1:100). PD-L1 was assessed as negative, weak positive which was defined as a membranous or cytoplasmic PD-L1 expression in 1 to 49% of tumor cells, and PD-L1 strong positive was defined as expression in ≥ 50% of tumor cells at higher power magnification. In this work, negative and weak positive stain was considered as a negative group (Aron et al. 2015).

### Data analysis

The analysis was performed using SPSS Statistics 23.0 (IBM Corp 2015). Data were summarized using descriptive statistics. Student’s *t* test was used to compare continuous data. The Kruskal-Wallis test was used instead as a nonparametric test. Logistic regression analysis was used to detect the independent factors in the ulcerative colitis.

## Results

### The prevalence of parasitic infections and Montreal’s classification of the UC patients

**Fig. 1:**
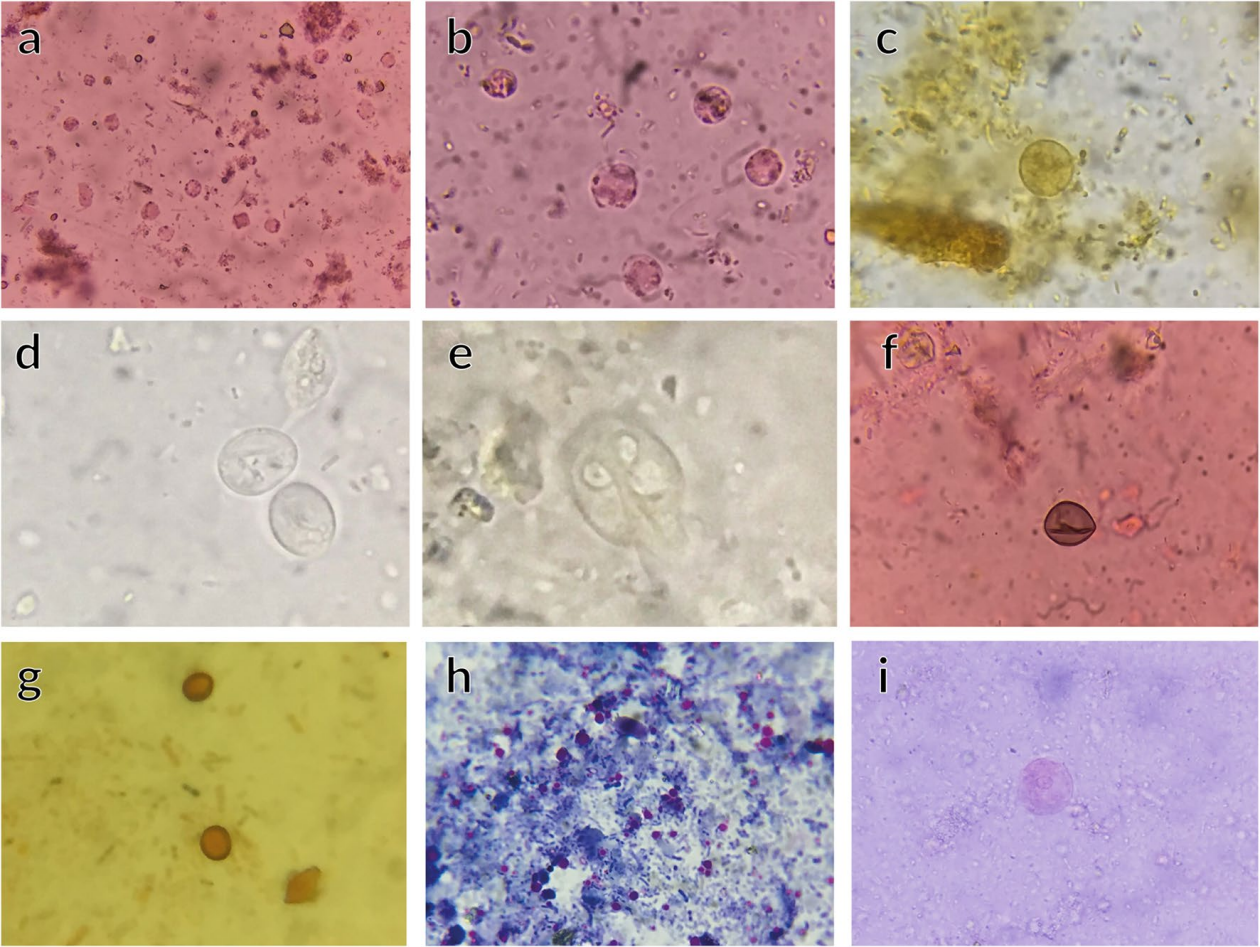
**a** *Blastocyst* vacuolar form eosin stain (×40). **b** *Blastocyst* vacuolar form eosin stain (×100). **c** *Blastocyst* vacuolar form iodine stain (×100). **d** *Giardia lamblia* cyst form wet mount (×100). **e** *Giardia lamblia* trophozoite form wet mount (×100). **f** *Giardia lamblia* eosin–stained cyst (×40). **g**–**h** *Cryptosporidium* iodine and Ziehl–Neelsen stain (×100). **i** *Entamoeba histolytica* trophozoite eosin stain (×100)

The most commonly identified parasites in GIII were *Blastocystis* in 52.33% of cases, followed by *Giardia lamblia*, *Cryptosporidium*, *Entamoeba histolytica/dispar,* and *Endolimax nana* in 20.56%, 14.95%, 6.54%, and 1.86% of cases, respectively. In keeping with Montreal’s classification of the UC patients (severity), in GIII, the percentage severity (S3) was 83.2%, while it was 31.1% in GII. The mean age of GIII was 37.1 ± 8.7, while that of GII was 44.0 ± 9.2 at the time of the index colonoscopy, with a statistically significant difference between both groups (*p* < 0.0001) (Table 1; Fig. 1).

**Table 1:** The demographic features, Montreal classifications, and prevalence of parasitic infections in the groups analyzed

Characteristics	Control individuals (*n* = 152)	UC patients without parasites (*n* = 45)	UC patients with parasites (*n* = 107)	*t*-test	*p*-value
Current age	44.2 ± 10.0	47.4 ± 9.8	45.1 ± 7.9	1.98	0.14
Gender					
Male	83 (54.6%)	20 (44.4%)	38 (35.5%)	*X*^2^ = 9.3	0.01**
Female	69 (45.4%)	25 (55.6%)	69 (64.5%)		
Age at diagnosis ± SD		44.0 ± 9.2	37.1 ± 8.7	4.4	0.000***
Montreal classification (extent)					
Procolitis E1		6 (13.3%)	9 (8.4%)	40.0	0.000***
Left sided E2		28 (62.2%)	16 (15.0%)		
Pancolitis E3		11 (24.4%)	82 (76.6%)		
Montreal classification (severity)					
Mild (S1)		9 (20.0%)	7 (6.5%)	39.8	0.000***
Moderate (S2)		22 (48.9%)	11 (10.3%)		
Severe (S3)		14 (31.1%)	89 (83.2%)		
Parasitic infection					
*Blastocystis* spp			56 (52.33%)		
*Giardia lamblia*			22 (20.56%)		
*Cryptosporidium*			16 (14.95%)		
*Entamoeba histolytica/dispar*			7 (6.54%)		
*Endolimax nana*			2 (1.86%)		
Co-infection with 2 parasites			4 (3.73%)		

**p-value < 0.01; ***p-value < 0.001

### The fecal and serological biomarkers

The FC values in GIII patients were noticeably more outstanding than in GI l. Serum CRP, IL-6, AOPP, MT, and p53Abs values were significantly elevated in patients with UC (GII and GIII), with considerably higher values in GIII than in those of GII. On the other hand, a significant decrease in serum GSH level was obtained in GII, with significantly lower values in GIII. There were statistically significant differences between the two groups (Table 2).

**Table 2:** Fecal and serological and biomarkers in UC patients infected and not infected with parasites

Investigations	Control individuals (*n* = 152)	UC patients without parasites (*n* = 45)	UC patients with parasites (*n* = 107)	*F*-test	*p*-value
Fecal Calprotectin (mg/kg)	23.3 ± 9.6	240.4 ± 158.56	1832.11 ± 1790.10^a^	KW = 95.5	0.000***
CRP (mg/L)	2.1 ± 0.8	5.6 ± 2.3^b^	8.0 ± 2.5^c,d^	326.4	0.000***
IL-6 level (pg/mL)	2.3 ± 0.8	3.0 ± 1.4^e^	5.3 ± 2.1^f,g^	135.6	0.000**
AOPP (μmol/L)	121.8 ± 21.2	145.1 ± 34.4^h^	199.1 ± 30.3^i,j^	266.0	0.000**
GSH (μmol/L)	2.8 ± 0.7	1.9 ± 0.6^k^	1.1 ± 0.9^l,m^	139.3	0.000**
MT (μg/mL)	5.2 ± 1.0	7.1 ± 1.1^n^	9.1 ± 1.5^o,p^	305.9	0.000**
p53Abs (U/mL)	9.3 ± 1.2	19.2 ± 2.8^q^	31.1 ± 3.6^r,s^	2260.5	0.000**

*KW* Kruskal-Wallis test

^a^Control vs group III: *p*-value = 0.000***

^b^Control vs group II: *p*-value = 0.000***

^c^Control vs group III: *p*-value = 0.000***

^d^Group II vs group III: *p*-value = 0.000***

^e^Control vs group II: *p*-value = 0.01**

^f^Control vs group III: *p*-value = 0.000***

^g^Group II vs group III: *p*-value = 0.000***

^h^Control vs group II: *p*-value = 0.000***

^i^Control vs group III: *p*-value = 0.000***

^j^Group II vs group III: *p*-value = 0.000***

^k^Control vs group II: *p*-value = 0.000***

^l^Control vs group III: *p*-value = 0.000***

^m^Group II vs group III: *p*-value = 0.000***

^n^Control vs group II: *p*-value = 0.000***

^o^Control vs group III: *p*-value = 0.000***

^p^Group II vs group III: *p*-value = 0.000***

^q^Control vs group II: *p*-value = 0.000***

^r^Control vs group III: *p*-value = 0.000***

^s^Group II vs group III: *p*-value = 0.000***

### The univariate and multivariate analyses of the groups studied

Using univariate and multivariate analysis, a statistically significant difference in fecal calprotectin and serum levels of CRP, IL6, AOPP, MT, and P53 in GIII were obtained compared to GII. When the GHs are considered, readings in GIII are statistically significantly lower than in GII. Highly statistically significant CRP readings in GIII were 2.3 times higher than in GII. AOPP, MT, and P53 measurements in GIII were 54.3, 1.9, and 11.9 times higher than in GII, respectively. IL-6 levels in GIII were 1.4 times higher than in GII, with statistically significant differences. Fecal calprotectin measurements in GIII were 1609 times higher than in GII, with vital statistical significance. The difference was highly statistically significant except for GSH, which was −0.7 times lower in GIII than in GII (Table 3).

**Table 3:** A univariate and multivariate analysis of UC patients infected and not infected with parasites

	GII vs GIII				
	Univariate analysis		Multivariate analysis		
	*F*-test	*p*-value	*F*-test	*p*-value	OR (95% CI)
CRP	20.3	0.000***	28.2	0.000****	2.3 (1.5 to 3.2)
IL6	8.1	0.005**	14.9	0.000****	1.4 (0.7 to 2.2)
Fecal calprotectin	25.4	0.000****	34.7	0.000****	1609(1069.2 to 2149.7)
AOPP	82.6	0.000****	89.7	0.000****	54.3 (42.9 to 65.7)
GSH	15.2	0.000****	18.9	0.000****	−0.7(−1.0 to 0.4)
MT	47.5	0.000****	56.9	0.000****	1.9 (1.4 to 2.4)
P53	302.0	0.000****	374.4	0.000****	11.9 (10.7 to 13.1)

*OR* odds ratio, *95% CI *95% confidence interval

**highly significant <0.01, ***very high significant <0.001

### The histopathological and immunohistochemical studies of the individuals

**Fig. 2:**
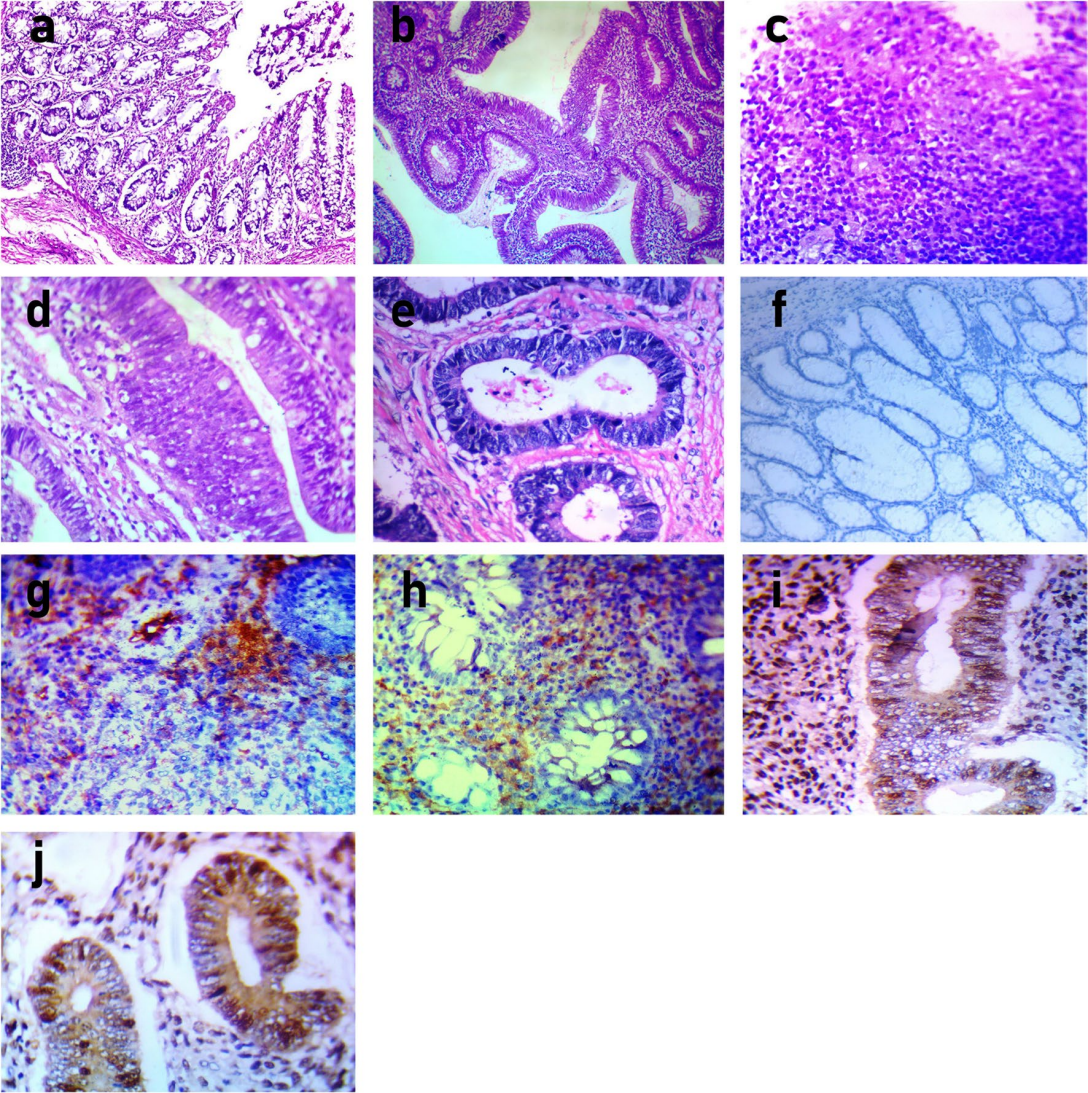
Photomicrograph of H&E and immunostained photomicrograph of programmed death ligand (PDLI) and P53 from colonic biopsy (×400). **a** GI with normal colonic mucosa. **b** GII with moderate inflammatory infiltrate with marked crypt distortion. **c** GIII with sever inflammatory infiltrate and marked mucosal ulceration. **d** GII with moderate dysplastic changes (nuclear hyperchromatism and stratification). **e** GIII with sever dysplastic changes (marked nuclear hyperchromatism and pleomorphism). **f** GI with negative PDL1 expression. **g** GII with moderate PDLI expression showing loss of membranous and cytoplasmic expression in colonic epithelium and moderate increased infiltration by the PDL1-positive cells in lamina propria. **h** GIII with strong PDLI expression showing loss of membranous and cytoplasmic expression in colonic epithelium and marked increased infiltration by the PDL1-positive cells in lamina propria. **i** GI with negative P53 expression. **j** GII with moderate dysplastic changes showing moderate P53 expression. **k** GIII with sever dysplastic changes showing strong P53 expression

Histopathological examination indicated severe inflammatory infiltration with significant mucosal ulceration in GIII (Fig. 2c) and a moderate inflammatory infiltrate with significant crypt distortion in GII (Fig. 2b), compared to normal colonic mucosa in GI (Fig. 2a). Moreover, mild dysplastic alterations (nuclear hyperchromatism and stratification) in GII (Fig. 2d) and severe dysplastic changes (marked nuclear hyperchromatism and pleomorphism) in GIII (Fig. 2e) were observed.

Immunostaining photomicrographs of PDLI and P53 colonic biopsies revealed significant PDLI expression with loss of membranous and cytoplasmic expression in colonic epithelium and considerable infiltration by PDL1-positive cells within the lamina propria in GIII (Fig. 2h). In GII, there was a moderate increase in PDL1-positive cell infiltration in the lamina propria (Fig. 2g) compared to negative PDL1 expression in GI (Fig. 2f). There was strong P53 expression in Fig. 2j and moderate P53 expression in Fig. 2i.

## Discussion

This study intended to explore the possible link between the inflammatory and dysplastic alterations in ulcerative colitis and intestinal protozoal infections. A positive association between parasitic infection and dysplastic changes in UC patients has been noted. Furthermore, Mostafa et al. (2018) confirmed the significant dysplastic alterations in the intestines of *Cryptosporidium*-infected mice. Moreover, CRC has been linked to an increased risk of *Cryptosporidium* spp. and *Blastocystis* spp infections (Taghipour et al. 2022).

One hundred seven of the 152 ulcerative colitis patients had parasite infections compared to controls. The most prevalent parasites were *Blastocystis* spp, *Giardia lamblia*, *Cryptosporidium*, *Entamoeba histolytica*/*dispar*, and *Endolimax nana*. These findings are consistent with those of Pestechian et al. (2021), who found a high prevalence of pathogenic intestinal protozoa in UC patients. In addition, *B. hominis* was detected more frequently in UC patients (Cekin et al. 2012). Our findings were significant and consistent with those of Toychiev et al. (2021), who confirmed the high prevalence of intestinal protozoa in ulcerative colitis and proposed anti-parasitic medication as a treatment option. In a case report, Pence et al. (2015) discovered that *B. hominis* may have a pathogenic effect.

According to the Montreal classification, S3 was found in 83.2% of GIII patients and 31.1% of GII patients. Iyer et al. (2013) confirmed that parasites had a role in the exacerbation of UC. These findings might be explained by the fact infection with bacterial, viral, or parasitic agents alters gut flora, prompting the emergence of chronic inflammatory and UC problems. In our research, GIII had an intense inflammatory infiltrate with significant mucosal ulceration, whereas GII had a moderate inflammatory infiltrate with significant crypt deformation. Parasitic infections appear to exacerbate inflammatory biomarkers in UC patients. Highly statistically significant increases in fecal calprotectin, serum levels of IL-6, and CRP were obtained in GIII compared to GII. The disease activity in Crohn’s disease (CD) and UC patients has been connected with serum levels of IL-6, soluble interleukin-2 receptor (sIL-2R), CRP, and FC values (Mavropoulou et al. 2020). Our findings may be explained by the fact that parasite protozoa change the structure of intestinal mucus and produce mucolytic enzymes, allowing their penetration of the mucus barrier and causing severe, prolonged inflammation (Tailford et al. 2015).

Continuous inflammation may lead to a preneoplastic event (Guina et al. 2015). The presented research obtained highly statistically significant increases in AOPP levels. Our findings are consistent with a prior study that found a rise in AOPP production in inflammatory bowel diseases (Krzystek-Korpacka et al. 2008) and that colon cancer is related to oxidative stress and elevated AOPP levels (Zińczuk et al. 2020). In addition, intestinal parasites, especially *Blastocystis* infection, were associated with a substantial oxidative burst, which resulted in oxidative stress (Chandramathi et al. 2010). Our findings show a significant drop in GSH levels in UC patients, with much lower levels in parasite-infected individuals. These results are in line with earlier research, which also demonstrated that GSH system depletion in UC patients causes increased susceptibility to carcinogenic compounds (Scibior et al. 2008). Patients with parasite infections may have reduced levels of GSH, which may encourage an accumulation of free radicals, which in turn cause damage to cell membranes and cellular components (RNA and DNA), resulting in neoplasia. Guevara-Flores et al. (2017) confirmed that in parasites, the enzymes involved in glutathione production and the GSH-dependent antioxidant mechanism are lacking.

Serum p53Ab values in both UC groups were considerably elevated in the current investigation, with significantly higher levels in patients with parasite infections (GIII). p53 is crucial for intestinal type 2 immunity in response to parasite infection (Chang et al. 2021). The commonly accepted concept for elevated serum p53Abs is that a point mutation in the p53 gene causes p53 protein overexpression, causing the release of p53Abs (Yoshizawa et al. 2007). Serum p53 autoantibody levels are used to determine the clinicopathological significance of gastric cancer (Oshima et al. 2020). Moreover, ROS modifies the p53 suppressor gene expression that has a critical role in apoptosis. Consequently, oxidative stress alters gene expression, cell proliferation, and apoptosis, and it is implicated in tumor genesis and development (Barrera 2012). Severe dysplastic alterations confirmed the importance of P53 expression in the colonic mucosa of GIII in our study compared to moderate P53 expression in GII. In our data, the significant rise of MT levels in both UC groups, with higher values in cases with parasitic infections, is consistent with the findings of Tsuji et al. (2013), who found an increase in MT expression in intestinal epithelial cells in IBD. It has been proven that variations in MT expression can be linked to carcinogenesis and cancer development (Krizkova et al. 2018). The probable function of MTs can explain our findings in the immune system and inflammatory processes and shield cells from free radicals and oxidative stress (McNeill et al. 2019). In addition, MTs are indirect regulators of Zn-dependent protein production, such as p53, which is essential for cell function (Bao et al. 2010).

The significant PDL1 expression in GIII in the presented research indicated advanced dysplastic alterations compared to the moderate PDL1 expression in GII. In various tumor types, PD-L1 expression can be controlled by extrinsic and intrinsic signals, including epigenetic modifications, chromosomal alterations, oncogenic and tumor suppressor signals, inflammatory cytokines, and other factors at the genetic, transcriptional, post-transcriptional, translational, and post-translational levels. CNAs on chromosome 9p affecting PD-L1 were identified in colorectal carcinomas (Budczies et al. 2016).

## Conclusion

The current study demonstrates a high frequency of intestinal protozoa, particularly waterborne ones, among UC patients. This results in oxidative stress and oxidative cellular damage, which are critical players in the etiology of the disease and the associated carcinogenetic process. Consequently, UC patients’ clinical prognosis can be improved by early screening for parasitic infections for early diagnosis and treatment of protozoan infections. Although our findings suggested a link between parasitic infections and the severity of dysplastic changes in ulcerative colitis patients, we plan to conduct larger research using parasitological molecular diagnostic techniques and genetic profiles of pro-inflammatory cytokines and dysplastic markers in the future to better understand the potential role of intestinal parasites. This could pave the way for a new therapy option for UC patients, perhaps alleviating a potentially fatal condition.

## Data Availability

Available with the corresponding author when requested

## Abbreviations

*UC*: ulcerative colitis
*FC*: fecal calprotectin
*p53Abs*: anti-p53 antibodies
*MTs*: metallothioneins
*GSH*: glutathione
*AOPPs*: advanced oxidation protein products
*CRP*: serum C-reactive protein
*IL*: interleukin
*IBD*: inflammatory bowel disease
*IRB*: Institutional Review Board
*C. parvum*: *Cryptosporidium parvum*
*B. hominis*: *Blastocystis hominis*
